# Maximizing Cannabinoid and Polyphenol Extraction from Industrial Hemp (*Cannabis sativa* L. cv. Helena) Areal Parts: A Comparative Study of Ultrasound-Assisted and Conventional Methods at Two Harvest Stages

**DOI:** 10.3390/plants14050816

**Published:** 2025-03-05

**Authors:** Zorica Lazarević, Anamarija Koren, Tijana Zeremski, Aleksandra Mišan, Nataša Nastić, Nadežda Stojanov, Senka Vidović

**Affiliations:** 1 Department of Pharmaceutical Engineering, Faculty of Technology, University of Novi Sad, 21000 Novi Sad, Serbia; zdrinic@mocbilja.rs (Z.L.); natasa.nastic@uns.ac.rs (N.N.); 2 Institute for Medicinal Plant Research ‘Dr. Josif Pančić’, 11000 Belgrade, Serbia; 3 Department of Organic Production and Biodiversity, Institute of Field and Vegetable Crops, 21000 Novi Sad, Serbia; anamarija.koren@ifvcns.ns.ac.rs (A.K.); tijana.zeremski@ifvcns.ns.ac.rs (T.Z.); nadezda.stojanov@ifvcns.ns.ac.rs (N.S.); 4 Institute of Food Technology in Novi Sad, University of Novi Sad, 21000 Novi Sad, Serbia; aleksandra.misan@fins.uns.ac.rs

**Keywords:** hemp, NBCs, cannabidiol, tetrahydrocannabinol, polyphenol, ultrasound-assisted extraction

## Abstract

In this work, two extraction techniques, conventional and ultrasound-assisted extraction (UAE) techniques, were employed for the extraction of natural bioactive compounds (NBCs) from the areal parts of industrial hemp (*Cannabis sativa* L. cv. Helena) at two harvesting stages: (i) the beginning of flowering and (ii) the full flowering of the hemp plants. In the conventional extraction, the effect of different extraction solvents on the extraction yield and the content of NBCs was examined. The extraction temperature, extraction time, and ultrasonic power were chosen for the process parameters in UAE. The highest value of the investigated responses in UAE-obtained extracts was higher compared to extract obtained with conventional extraction techniques when the same solvent was used (50% ethanol): extraction yield (17.54 compared to 15.28%), content of total phenols and total flavonoids (1.7795 compared to 1.0476 mg GAE/mL and 0.6749 compared to 0.3564 mg CE/mL, respectively) and cannabidiol (0.8752 compared to 0.4310 mg/mL). Comparing the plant material in different developmental stages, it can be concluded that hemp aerial parts at the beginning of the flowering stage represent a good source of the phenolic compound with sinapic acid and apigenin being dominant, while hemp aerial parts in the full flowering stage represent a good source of cannabinoids.

## 1. Introduction

*Cannabis sativa* L., recognized under the term industrial hemp, is a plant with a long tradition of growth and a wide tradition of usage, covering applications in different industrial sectors, starting from food and pharmaceutical to construction and textile production. Up to now, cultivation has mostly occurred for two high-value products: fiber and seeds. The fiber hemp is applied in the paper, textile, and construction industries, while the seed hemp is mostly used for the production of cold-pressed seed oil, and further flour and proteins. In addition to fiber and seeds, aerial parts of hemp, including leaves and flowers, are of importance as they contain biologically active compounds, such are polyphenols and cannabinoids. Although new non-cannabinoid and cannabinoid constituents are frequently discovered, the most commonly reported compounds in *C. sativa* L. are cannabinoids, non–cannabinoid phenols, flavonoids, terpenes, alkaloids, and others [1,2]. Cannabinoids represent the most studied group of the *C. sativa* L. constituents due to their wide range of bioactive effects [3]. According to Odieka et al. [1], the most important cannabinoids found in hemp are tetrahydrocannabinol (Δ^9^-THC), cannabidiol (CBD), and cannabinol (CBN), followed by cannabigerol (CBG), cannabichromen (CBC), and cannabinodiol (CBNDA). CBD is defined as a major cannabinoid present in hemp [3]. A recent study conducted by Radwan et al. [2] resulted in the identification of forty-two non-cannabinoid phenols (spiro-indans, dihydrostilbenes, dihydrophenathrenes, and simple phenols) and thirty-four flavonoids (C-glycoside and O-glycoside of orientin, vitexin, isovitexin, apigenin, luteolin, kaempferol, and quercetin) have been detected in *C. sativa* L.

It has been reported that identifying the optimum harvesting stage of *C. sativa* is significant because the plant growth stage affects the chemical composition [4,5]. Therefore, it can be concluded that the chemical profile will change during plant development and that the composition of phenolics and cannabinoids in hemp will be different in immature and mature plants. Meijer et al. [6] reported that CBC content is present in large amounts in immature plants and that its content decreases with plant maturation. The content of other cannabinoids, such as THC, increases with maturation, and it is the highest in the budding stage [4,5].

The biological activities of the hemp are linked to its chemical constituents. According to Burstein [7], CBD, the dominant cannabinoid of hemp, is showing a range of biological activities such as: anti-arthritic, anti-psychotic, anti-anxiety, anti-nausea, anti-inflammatory, and immunomodulatory. Hill et al. [8] reported that CBD is a potentially promising therapeutic agent for different central nervous system diseases, including epilepsy, neurodegenerative diseases, schizophrenia, multiple sclerosis, affective disorders, and others. In addition, it has shown both antimicrobial and antifungal effects [9]. Also, it is now well-established and documented that CBD is able to reduce the side psychoactive effects of THC [10]. Flavonoids present in hemp have anti-inflammatory, anticancer, and neuroprotective effects [11].

For the isolation of these biologically active constituents, various extraction techniques can be applied, such as conventional maceration or Soxhlet extraction, or advanced and green techniques, starting from high-pressure techniques, such as supercritical fluid extraction and subcritical water extraction, to extraction techniques such as enzyme-assisted, microwave-assisted, or ultrasound-assisted. The selection of the most appropriate extraction technique, as well as the optimal set-up of process parameters, are the key factors that will enable the efficient recovery of the targeted bioactive compounds. Both the applied technique and the process parameters set-up are dependent on the nature and the sensitivity of compounds targeted for extraction. Therefore, knowledge of the impact of dominant process parameters on the efficiency of the extraction of target compounds in each selected extraction technique is needed. In addition to process efficiency, the extraction techniques selected should also be evaluated by considering the time required for the completed extraction, as well as energy consumption, because both will affect the further application of such process on the industrial level.

In a conventional extraction process, parameters such as time, solvent, solvent-to-drug ratio, particle size of herbal material, and temperature can be varied. According to Picot-Allain et al. [12], conventional extraction methods have shortcomings, such as low extraction rate, large solvent consumption, long time of extraction, and high energy consumption, which can be overcome by the application of the advanced techniques. Among the advanced techniques, ultrasound-assisted extraction (UAE) is recognized as one of the most efficient, inexpensive, and simple existing extraction systems working with a reduced amount of solvent, which is especially suitable for the isolation of moderate- and high-polar compounds [13,14,15]. The UAE is affected by various factors, such as extraction temperature, extraction time, ultrasonic power, solvent properties, composition, particle size, and solid-to-solvent ratio [16]. According to Siddique et al. [17], in the UAE, applied ultrasonic waves cause various physical and chemical phenomena, such as cavitation, agitation, vibration, pressure, shock waves, friction, microturbulence, compression, and expansion, which are responsible for the increased mass transfer.

Hemp extraction can be divided into two categories, depending on the constituent or fraction targeted that needs to be isolated: extraction of the cannabinoids, terpenes, and phenolics from aerial parts and inflorescences, and the extraction of oil fractions from the hemp seeds. The extraction of oil fractions from hemp seeds is usually provided using conventional cold-pressing. The extraction of cannabinoids and phenolics, so far, has been done with different extraction techniques: conventional and modified maceration [18,19,20], Soxhlet extraction [20,21], solid-phase extraction [22], supercritical fluid extraction [20,22], microwave-assisted extraction [23], and UAE [24].

Considering all the information given, the aim of the following study was twofold: (i) to investigate the composition of natural bioactive compounds (NBCs), polyphenols, and cannabinoids, which are present in the aerial parts of the industrial hemp cultivar, Helena, including their variations in composition in the plant at the beginning of, and in the full flowering stage, and (ii) to examine the preparation of hemp herbal extracts and the potential of extraction techniques—the conventional and UAE for the isolation of these constituents. In conventional extraction, special research attention has been directed toward the effect of extraction solvent (water/ethanol mixtures and pure water) on the extraction yield of targeted compounds, while in the UAE, it has been directed toward the impact of parameters, such are extraction temperature, extraction time, and ultrasonic power in the selected range.

## 2. Results and Discussion

### 2.1. Impact of the Plant Stage Growth and Applied Solvent on the Profile of Phenolic Compounds, CBD and THC in Hemp Extracts

During plant growth, different NBCs are generated and accumulated in different parts of the plant. Hence, for crop valorization, it is of great importance to harvest plants in the proper stage of growth—the stage characterized by the highest concentration of the NBCs of interest. Many authors have reported age-dependent variations in the qualitative phenolic composition of different plants. Chepel, Lisun, and Skrypnik [25] reported that total phenol content (TP) in Heather was different throughout different growth stages and that the maximum value for TP was found in the growth stage just before flowering. *Portulaca oleracea* leaves and steam have the highest total phenolic content at the mature plant stage [26], while *Trifolium pratense* have higher amounts of phenolic compounds at the lowest growth stage [27]. Cobaleda-Velasco et al. [28] reported that the phenolic profiles of *Physalis angulata* were qualitatively and quantitatively different during different growth phases. Therefore, the focus of the study was directed toward the two growth stages of hemp: at the beginning of flowering and in the full flowering stage. In addition, the impact of the nature of the material in need of extraction, the extraction yield, and the content of the NBCs in extracts are also dependent on the solvent applied for the extraction process [29]. Properly selecting the type of extraction solvent enables the production of herbal extracts rich in NBCs. According to several authors, methanol, ethanol, acetone, and their mixtures with water are reported as extraction solvents that can be applied for the extraction of NBCs like phenolics [29,30]. Bahorun et al. [31] and Durling et al. [32] characterized the water/ethanol mixtures as the best solvent combination for the extraction of phenolic compounds. According to the research of Mkpenie et al. [33], water/ethanol mixtures, with an ethanol concentration between 30% and 60%, were found to give the highest extraction yields of phenolic compounds like rosmarinic and caffeic acid. The scientific data also show the fact that the increase in water content in the mixture of water and other organic solvent leads to the general increase in the extraction yield [29,30], mainly due to the increase in concomitant constituents.

Considering the scientific data, the study investigated the efficiency of water and water/ethanol solutions for applications in the extraction of phenolic compounds from the hemp areal part at both growth stages. From an effective extraction process standpoint, in addition to the extraction of phenolic compounds (TPs and total flavonoids (TFs)), general extraction yield (EY) was observed as well.

The EY ranged from 8.18% to 17.94% and from 7.54% to 15.28% for aerial parts at the beginning of the flowering stage and in the full flowering stage, respectively (Table 1). In comparison to the yields obtained the areal parts of hemp in the full flowering stage, higher yields were observed for all cases of extraction of areal parts at the beginning of the flowering stage, regardless of which solvent was applied. The EY increased with increased water content in applied water/ethanol mixtures with a statistically significant difference, in accordance with observation of several authors [29,30]. The most efficient solvent with respect to general EY for both hemp samples was pure water, and this can be explained by the lowest selectivity of water at the room temperature.

TP and TF content were measured in the extracts obtained from both samples of hemp, which are shown in Table 1. The extracts obtained from the aerial parts of hemp at the beginning of the flowering TPs were higher, ranging from 0.5759 to 1.7045 mg GAE/g mL, in comparison to the TPs of the extracts obtained from the aerial parts of hemp in the full flowering stage where they ranged from 0.4996 to 1.0476 mg GAE/mL. For both samples, the extracts of the lowest TP content without a statistically significant difference were detected in the case where water and 90% ethanol were applied as an extraction solvent. This can be explained by the high polarity of water, which is not compatible with the polarity of the phenolics present in the investigated material. In the case of material in the full flowering stage, the highest TP concentration was achieved when 50% ethanol was applied as an extraction solvent, although there was no statistically significant difference even when 70% and 30% ethanol were used. This is in accordance with the observation of several authors who reported that 50% ethanol is the solvent with higher extraction efficiency toward phenolic compounds in comparison to other water/ethanol mixtures [34,35]. The highest TP extraction (1.7045 mg/mL) was achieved via the application of 70% ethanol in the case of the hemp, which was at the beginning of the flowering stage; this content was up to 40% higher in comparison to the highest achieved hemp-use in the full flowering stage, which could implicate that plants at the beginning of the flowering stage can be more appropriately used for the isolation of TPs, but the detailed profile of individual phenolic constituents should be considered before the final conclusion is made.

The TF concentration in the obtained extracts ranged from 0.2370 to 0.4817 mg CE/g mL and from 0.1674 to 0.3584 mg CE/mL for the aerial parts of hemp at the beginning of the flowering stage and in the full flowering stage, respectively. In both cases, the highest TF content was obtained by applying 50% ethanol as an extraction solvent, with a statistically significant difference in the TF content of the extracts obtained from other solvents used. In parallel to the case of TPs, the lowest content of TFs in the extracts with statistically significant differences for both materials was observed in the case where pure water was applied. In previous research, Mkpenie et al. [33] compared the composition of ethanol, methanol, and acetone hemp leaf extracts, and found that ethanol, applied as an extraction solvent, gave the best results in terms of polyphenol extractions. The same authors also reported that polyphenols present in the hemp leaves were of moderate polarity. This is in agreement with the highest TP and TF concentrations obtained via 50% ethanol from the areal parts of hemp in the full flowering stage and is in agreement with the lowest contents measured in the extracts from the same material produced by the water.

Qualitative and quantitative profiles of individual phenolic compounds detected in the obtained hemp extract assortment of Helena are given in Table 2.

Eleven different phenolic compounds were detected in the investigated extracts obtained from the areal parts of the hemp assortment of Helena. These are as follows: protocatechuic acid, vanillic acid, syringic acid, epicatechin, ferulic acid, sinapic acid, isovitexin, rutin, cinnamic acid, naringenin, and apigenin. In all the hemp extracts obtained from the areal parts at the beginning of the flowering stage the following phenolics were found regardless of the extraction solvent applied: protocatechuic acid, ferulic acid, sinapic acid, cinnamic acid, and apigenin. In the case of the extracts obtained from the areal parts in the full flowering stage, only naringenin and apigenin were present in all extracts, regardless of the extraction solvent applied. Ferulic was absent in the extracts of hemp in the full flowering stage, while syringic acid and epicatechin were present only in the one produced by 70% ethanol.

Generally, sinapic acid was the most dominant phenolic present in the investigated hemp extracts. The higher concentrations of sinapic acid were detected in the extracts produced from the areal parts of the hemp at the beginning of the flowering stage (from 1.13 to 30.02 µg/mL) in comparison to those produced from the material in the full flowering stage, except in the case of those produced by water. The highest concentration was measured in the hemp at the beginning of the flowering stage; the extract was prepared by using 50% ethanol. Several biological activities have been ascribed to sinapic acid, with antimicrobial, anti-inflammatory, anticancer, and antianxiety being among them [36]. In accordance with our findings, a decrease in sinapic acid concentration during the maturation of *Mespilus germanica* fruit was reported by Gruz et al. [37]. This group of authors reported that the decrease in free esters in phenolic acids is related to their binding to cell walls, their transformation into compounds that are no longer detectable by HPLC, and a reduction in primary metabolism during maturation.

Second, the dominant compound in the hemp extract was naringenin. In total, 30% ethanol was the most efficient extraction solvent for the isolation of this compound. According to the results, naringenin is present in the areal parts of hemp at the beginning of the flowering stage, while its concentration decreases during plant maturation. Therefore, the measured concentration of this hemp phenolic in the extract obtained from the material in the full flowering stage using the same solvent (30% ethanol) is approximately 30 times lower in comparison to the content in the extract produced from the areal hemp parts at the beginning of the flowering stage. Studies have reported that naringenin exhibits antioxidant, antitumor, antiviral, antibacterial, anti-inflammatory, antiadipogenic, and cardioprotective effects [38]. Choi et al. [39] reported that the content of naringenin in the peel extracts of immature citrus fruits was 1.67 mg/g, while in the peel extracts of mature fruits, it was not detected, which is in accordance with our study, which indicates that the content of naringenin decreases with plant maturation.

Syringic acid and epicatechin were present in the extracts obtained using 70% ethanol for hemp areal parts at the beginning of the flowering stage and in the extracts obtained using 70% ethanol and water for hemp areal parts in the full flowering stage. The content of syringic acid was approximately 3-fold higher in the extracts obtained from the hemp areal parts in the full flowering stage (4.77 µg/mL), while the content of epicatechin was approximately 2-fold lower (3.59 µg/mL) in comparison to the content produced when hemp areal parts at the beginning of flowering stage were used (7.384 µg/mL). This can indicate that the concentration of syringic acid in the hemp areal parts is increasing during the growth and development of the plant, while this is not the case with epicatechin.

Ferulic acid was present in all the extracts obtained from the hemp areal parts at the beginning of the flowering stage, while it was not detected in the extracts obtained from the material in the full flowering stage, meaning that this phenolic component decreases during plant maturation. The highest concentration of the ferulic acid (3.61 µg/mL) was measured in the extract prepared using 50% ethanol as an extraction solvent.

According to the obtained results, 70% ethanol was the most efficient extraction solvent for the isolation of cinnamic acid from the hemp in the full flowering stage, where the higher concentration of this phenolic compound was detected in comparison to the plant material at the beginning of the flowering stage. This is in accordance with a previously reported study that hydroxycinnamic acids increase during maturation to the flowering stage and then decrease at the stage of seed ripening [25]. The highest concentration of this acid was 8.84 µg/mL. Cinnamic acid has shown a wide range of biological activity: antioxidant, antimicrobial, anticancer, neuroprotective, anti-inflammatory, and antidiabetic properties [40].

The concentration of isovitexin was the highest (6.21 µg/mL) in the extract that was also obtained from the material in the full flowering stage, using 50% ethanol as extraction solvent. Using the same solvent, 50% ethanol, the most efficient extraction of apigen was achieved. In the obtained hemp extracts, the concentration of apigenin was generally higher in the extracts produced from the hemp areal parts at the beginning of the flowering stage, ranging from 0.31 to 8.81 µg/mL, while its concentration in the extracts produced from the areal parts in the full flowering stage was low, close to 1 µg/mL. Based on the obtained results, it can be concluded that apigenin is more characteristic for plants at the beginning of the flowering stage, while its concentration decreases during plant maturation, similar to the case of naringenin.

According to Mahlberg and Kim [41], cannabinoids are formed through the condensation of terpene and phenol precursors. It has been reported that phenols are transported as glycosides to the vacuole, where their aglycone component enters the cell. Phenols in the aglycone form are released from the vacuole, accumulate in the plasma, and form cannabinoids with terpenes. In regard to the previously mentioned, it can be concluded that the lower content of some phenolic compounds in the plant material in the full flowering stage and their corresponding extracts can be caused by the biosynthetic pathway of the cannabinoids. This also explains the higher quality of the extracts obtained from the areal hemp parts at the beginning of flowering, which are in the mean variety, and the quantity of phenolic compounds present.

Izzo et al. [42] examined phenolic compounds in different commercial hemp inflorescences. They reported that hydroxycinnamic acids (chlorogenic acid, caffeic acid, p-coumaric acid, and ferulic acid), lignanamides (cannabisin A, B, C), phenolic amides (n-trans-caffeoyltyramine), flavonols (rutin, quercetin-3-glucoside, kaempferol-3-O-glucoside, quercetin, and kaempferol), flavones (cannflavin A and B, luteolin-7-O-glucoside, apigenin-7-O-glucoside, Luteolin, and apigenin), flavanols (catechin and epicatechin), and flavanone (naringenin) were found. The phenolic profile of the extracts obtained from hemp cv. Helene was similar to the phenolic profile of the different hemp varieties reported by Izzo et al. [42]. Slight differences can be related to the influence of biotic and abiotic factors that affect the biosynthetic process of the compounds studied [43].

Summarizing all the obtained results, it can be concluded that in the case of this way set extraction, 50% ethanol can be selected as the extraction solvent of first choice, especially in the case of plant materials harvested at the beginning of crop flowering, as it enables the production of extracts that are of the highest concentration of sinapic acid and apigenin. That is why in the same extracts obtained from both materials—at the beginning of the flowering stage and at the full flowering stage—the concentrations of CBD and THC were evaluated. According to the obtained results, the concentration of CBD was approximately 2-fold higher in the case of the extract produced from the material in the full flowering stage (0.431 mg/mL) in comparison to the concentration of CBD in the extract produced from the plant material at the beginning of the crop flowering (0.236 mg/mL). Contrary to this, the concentration of THC was lower in the extracts produced from the hemp areal parts at the full flowering stage (0.036 mg/mL), while in the extract produced from the hemp material at the beginning of the flowering stage, the concentration of THC was 0.0530 mg/mL. Cannabinoids are the most investigated compounds of hemp. For the extraction of these compounds, many solvents and extraction techniques were applied. The potential of ethanol to be applied for the extraction of these was also found in one of the previous investigations. Namely, Brighenti et al. [44] examined the application of different types of extraction solvents for the recovery of cannabinoids from the different samples of the hemp female inflorescence and found that ethanol gave the best results.

### 2.2. Ultrasound-Assisted Extraction (UAE) of Phenolic Compounds, CBD, and THC from the Areal Parts of the Hemp

Knowing the advanced possibilities of UAE, this study focused on the application of UAE for the extraction of hemp NBCs, just like the case of conventional extraction. Previously, the efficiency of the microwave-assisted extraction (MAE) of the same hemp materials’ areal parts from the industrial hemp cultivar, Helena, which targeted the same compounds, has been provided [23]. According to study on MAE, using this extraction technique, and depending on the parameters applied, it is possible to produce hemp extracts with EY between 10.96 and 38.08%, TP ranging from 0.8499 to 2.7060 mg GAE/mL, and content of TF ranging from 0.4707 to 1.4246 mg CE/mL, while cannabinoid content can be achieved, which ranges between 0.2243 and 1.8415 mg/mL for CBD and 0.0339 and 0.0637 mg/mL for THC [23]. Agarwal et al. [24] investigated UAE of NBCs, including polyphenols and flavonoids from the inflorescences of fiber-type hemp. In the Agarwal study, the influence of different process parameters, such as extraction time (5, 10, and 15 min), ultrasound power (90%, 120%, and 150%), and concentration of solvent (20%, 50%, and 80%) on extraction yield, total phenol content, total flavonoid content, and antioxidant capacity determined by FRAP was evaluated. The process was optimized using RSM. In the obtained extracts, the extraction yield was between 3.51 and 6.39%, the content of total phenols ranged from 9.47 to 15.70 mg GAE/mL, and the content of TF ranged from 0.84 to 2.55 mg QE/mL. Also, ultrasound extracts have a higher content of cannabinoids compared to the control one. The main cannabinoids identified were CBG and THC. This study did not evaluate the content of cannabinoids. The same authors reported that the EY, TP, and TF content were over twice as high in the extracts obtained by UAE in comparison to the ones produced via conventional extraction.

For the evaluation of UAE efficiency of NBCs from the areal parts of hemp cv. Helena, several process parameters were varied according to data given in Table 3. The obtained experimental results of investigated responses (EY, TP, TF, CBD, and THC) are also presented in Table 3. As the biological activities of industrial hemp are mainly related to cannabinoids, i.e., CBD, and the cannabinoids content, as mentioned in the introduction, through an increase during plant maturation, UAE optimization was performed only for the hemp aerial parts at the full flowering stage. The higher content of cannabinoids with hemp maturation was also confirmed in this study since hemp extract in the full flowering stage is approximately two-fold higher for CBD content.

Based on the results obtained for conventional extraction, 50% ethanol was chosen as the solvent in the UAE due to the highest content of phenolic compounds in the extract obtained with this solvent. In addition, ethanol in the concentration of 50% has been selected by many authors as a solvent that is highly efficient for the extraction of phenolic compounds [34,35,45]. A high content of phenolic compounds in extracts is desirable because the phenolic compounds have a synergistic effect with cannabinoids. Additionally, the advantages of ethanol as an extraction solvent are its low cost and non-toxicity for human consumption.

According to the results, the EY achieved in the UAE experiment ranged from 10.00 to 17.54%. The highest yield was obtained for the following conditions: extraction temperature—60 °C, extraction time—60 min, and ultrasonic power density—42 W/L. In the extracts obtained by UAE, TP ranged from 1.0552 mg GAE/mL to 1.7795 mg GAE/mL. The highest TP was obtained for the following conditions: extraction temperature—80 °C, extraction time—60 min, and ultrasonic power density—42 W/L. TF content ranged from 0.3793 mg CE/mL to 0.6749 mg CE/mL, where the extract with the highest value of TF, 0.6749 mg CE/mL, was obtained under the following conditions: extraction temperature—60 °C, extraction time—60 min, and ultrasonic power density—60 W/L. Regarding cannabinoid content, THC content ranges from 0.0412 mg/mL to 0.0458 mg/mL, while the content of CBD was higher, ranging from 0.6158 mg/mL extract to 0.8752 mg/mL. The conditions which enabled the preparation of the extract of the highest investigated cannabinoid content were an extraction temperature of 80 °C, an extraction time of 40 min, and an ultrasonic power density of 60 W/L.

The response surface methodology (RSM) and artificial neural networks (ANNs) are the most frequently used statistical approaches for modeling and optimization for the extraction of bioactive compounds from plant material.

In the case of RSM, the values obtained for each of the responses investigated were fitted into a second-order polynomial, and the model fitting was checked through variance analysis (ANOVA). The coefficient of determination (R2), coefficient of variance (CV), and *p* value for the model and lack-of-fit testing are summarized in Table S1. The coefficient of determination was used as the first parameter for model-fitting checking. The coefficients of determination were relatively high (above 0.8) for all investigated responses. Although R2 in the designed experiment was not high (close to 1), the mathematical models were statistically acceptable due to the highly significant regression for the model (pm < 0.05) and non-significant lack-of-fit (*p* > 0.05), meaning the dispersion of experimental data was a model-independent measure of pure error. The coefficient of variance is low (CV < 10%) in each model and supports good precision and reproducibility of the model. In general, all these statistical parameters suggest that the model that was used adequately represents the relationship between independent variables and different responses.

Based on the data given in Table S2, which presented corresponding *p* values of linear, interaction, and quadratic terms of the regression coefficient obtained for the selected response variables, the effect of each process parameter on each response can be evaluated. Based on these, mathematical equations, describing the impact of the parameters are generated and given in Table S3.

Regression analysis showed that temperature is the main factor which affects all outputs with a remarkably significant positive influence of the linear term (*p* < 0.01), meaning that by increasing the temperature, all outputs will increase. The quadratic term of the extraction temperature had a significant negative impact on the TF content, created an increase in the extraction temperature to a certain point, which led to an increase in TF content, and decreased the efficiency of the TF extraction. Ultrasonic power affected only the EY, and its linear term had a highly significant positive influence (*p* < 0.05), meaning that an increase in this parameter leads to an increase in the EY. The interaction of extraction temperature and time has a highly significant negative influence on TF and THC content (0.01 < *p* < 0.05), while on CBD content, it has a significantly negative influence (0.05 < *p* < 0.1). This means that with an increase in extraction time and extraction temperature, the content of TF, THC, and CBD will decrease.

The positive temperature influence on extraction yield and the content of investigated NBCs can be explained by increasing the solubility and diffusivity of these compounds, with a temperature increase [46]. Prolonged extraction times at higher temperatures can lead to a decrease in the extraction efficiency of some compounds due to their degradation. Generally, increasing ultrasonic power can lead to higher efficiency of UAE in terms of yield, with lower selectivity of target molecules [47,48].

**Figure 1:**
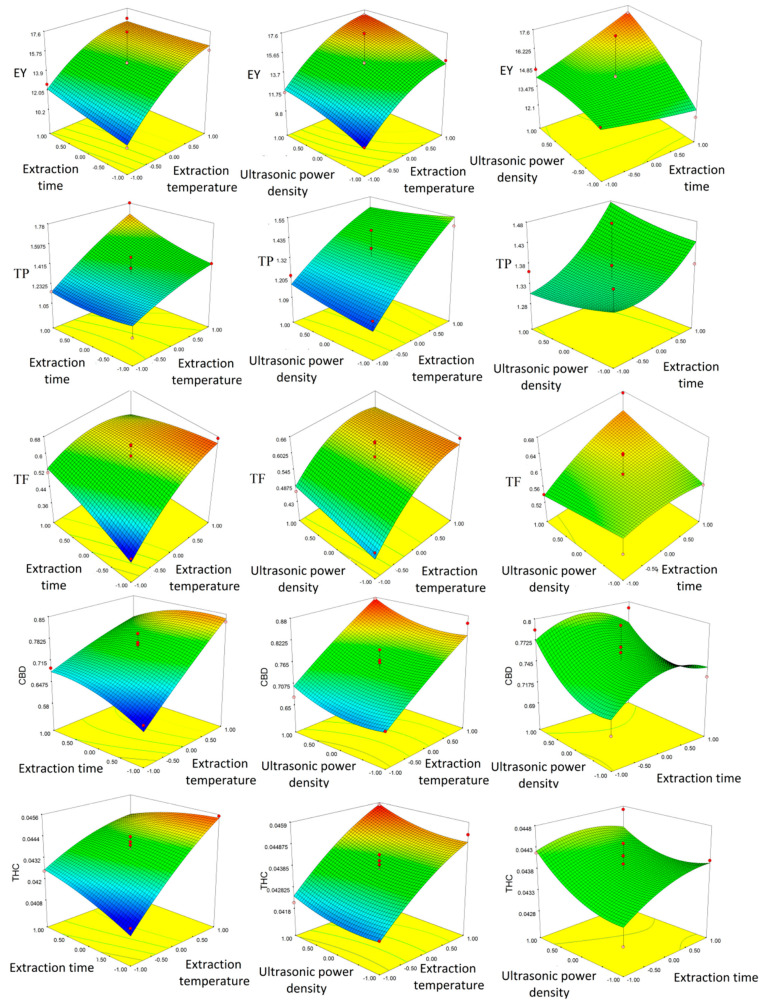
Three-dimensional plots obtained by response surface methodology (RSM) showing combined effects of parameters on extraction yield (EY), total phenol (TP), total flavonoid (TF), cannabidiol (CBD), and tetrahydrocannabinol (THC).

The same results can be seen from the 3D plots presented in Figure 1.

The optimization of UAE for all responses investigated was based on the experimental results and statistical analysis. The optimization was conducted to carry out the process in the most efficient way. Estimated optimal conditions for the investigated range and predicted values of investigated responses for all three optimizations are given in Table 4.

The optimal conditions for the areal hemp part extraction according to the maximum content of TP and TF are as follows: extraction temperature of 68.8 °C, extraction time of 54.8 min, and ultrasonic power density of 60 W/L. Applying these conditions, the predicted values of the investigated response were 17.54%, 1.5245 mg GAE/mL, 0.6214 mg CE/mL, 0.8077 mg/mL, and 0.0448 mg/mL for EY, TP, TF, CBD, and THC, respectively. The corresponding chromatograms are shown in Figure S1.

If the optimum was set in such a way as to obtain the hemp extract with the highest possible CBD content, then the following conditions should be applied: extraction temperature of 79.4 °C, extraction time of 31.6 min, and ultrasonic power density of 60 W/L. Using these conditions, the predicted values for EY, TP, TF, CBD, and THC content were 16.85%, 1.4435 mg GAE/mL, 0.6092 mg CE/mL, 0.8827 mg/mL, and 0.0460 mg/mL, respectively.

If the optimum was set in such a way as to produce hemp extract with the highest possible content of CBD and the lowest possible content of THC, then the following extraction conditions needed to be applied: an extraction temperature of 55.4 °C, an extraction time of 38.2 min, and an ultrasonic power density of 24 W/L. By setting these parameters, the predicted values for EY, TP, TF, CBD, and THC were 13.43%, 1.2370 mg GAE/mL, 0.5368 mg CE/mL, 0.7336 mg/mL, and 0.0431 mg/mL, respectively.

Validation was performed according to the optimal conditions obtained for the maximum content of TP and TF. The experimental values of the investigated responses were as follows: 18.46%, 1.5128 mg GAE/mL, 0.7895 mg CE/mL, 0.8565 mg/mL, and 0.0429 mg/mL for EY, TP, TF, CBD, and THC, respectively. The values of the investigated responses correspond to the predicted values with a 95% confidence interval.

On the defined optimal conditions by RSM for preparation extracts with the highest possible TPs and TFs, two extracts were prepared using the areal parts of the hemp at the beginning of the flowering stage and in the full flowering stage, based on an assortment of Helena. In this way, the prepared extracts’ individual phenolic compounds were identified and quantified. The results are given in Table 5 and Figure S2.

The most dominant phenolic compound in the produced extracts, in the case of hemp plant material from both harvest times, was sinapic acid. This is in accordance with the HPLC results obtained in the case of the hemp extract obtained using the conventional method of extraction. The extract of plant material at the beginning of the flowering stage had about a 2-fold higher content of sinapic acid compared to the extract obtained from the plant material in the full flowering stage (25.00 compared to 10.04 μg/mL). Naringenin and apigenin content were higher by about 3- and 2-fold, respectively, in the extract obtained from hemp at the beginning of the flowering stage (6.50 and 2.00 compared to 2.50 and 1.28 μg/mL, respectively), while isovitexin content was about 6-fold higher in the extract of hemp at the beginning of the flowering stage (6.56 compared to 1.04 μg/mL). The content of rutin was about 2-fold higher in the extract obtained from hemp in the full flowering stage (3.92 compared to 1.52 μg/mL), while the cinnamic acid content was slightly higher (2.04 compared to 1.55 μg/mL). Protocatechuic acid, vanillic acid, and ferulic acid content were similar in both extracts. According to the results obtained in both the applied extraction methods, conventional and UAE, it can be concluded that when the target compounds are phenols, the areal hemp parts should be harvested for extraction when the crop is at the beginning of flowering.

Comparing the two extraction methods, conventional and UAE, regardless of plant harvest time, it can be concluded that UAE favors the extraction of some phenolic compounds when the same solvent is used (50% ethanol). Higher content of protocatechin acid, vanillic acid, ferulic acid, sinapic acid, rutin, and naringenin was obtained when UAE was applied. The content of sinapic acid was about 10-fold higher in the UAE extract obtained from hemp in the full flowering stage than in the conventional extract (10.04 and 1.03 μg/mL, respectively), while in the conventional extract obtained from hemp at the beginning of the flowering stage, it was slightly higher compared to the UAE extract (30.02 and 25.00 μg/mL, respectively). Protocatechuic acid and ferulic acid were not within the detection limits in the extract obtained by using conventional extraction from hemp in the full flowering stage, while their content in the extract obtained by UAE was 1.28 and 2.22 μg/mL, respectively. The content of these two acids in both conventional and UAE extracts of hemp at the beginning of the flowering stage was similar (1.50 and 1.34 μg/mL for protocatechuic acid, and 3.61 and 3.36 μg/mL for ferulic acid, respectively). The contents of vanillic acid and rutin were slightly higher in the UAE extract in both used plant materials: 1.95 and 2.22 μg/mL in UAE extracts compared to 1.44 and 1.26 μg/mL in conventional extracts from hemp at the beginning of flowering stage and in the full flowering stage, respectively, and 1.52 and 3.92 μg/mL in UAE extracts compared to 0.19 and 2.74 μg/mL in conventional extracts from hemp at the beginning of flowering stage and in the full flowering stage, respectively. Higher content of isovitexin was obtained in UAE extract from hemp at the beginning of the flowering stage, while in the conventional extract obtained from hemp at the full flowering stage, the content of isovitexin was higher at the same rate, about 5-fold. Naringenin was not detected in the extract obtained via conventional extraction from material at the beginning of the flowering stage, while in UAE extract, its content was 6.50 μg/mL. The content of naringenin in UAE extract from hemp in the full flowering stage was slightly higher compared to conventional extract (2.50 compared to 1.33 µg/mL). Cinnamic acid had similar content in both extracts, conventional and UAE, obtained from hemp aerial parts at the beginning of the flowering stage (1.44 and 1.55 μg/mL, respectively), while in conventional extract of hemp aerial parts in the full flowering stage was higher than UAE extract (3.28 compared to 2.04 μg/mL, respectively). Apigenin content was about 4-fold higher in the conventional extract of hemp at the beginning of the flowering stage than UAE (8.81 compared to 2.00 μg/mL, respectively), while its content in both extract, conventional and UAE, obtained from hemp in the full flowering stage, was similar (1.10 and 1.28 μg/mL, respectively). In addition to the content of different phenols, comparing both extraction methods used for hemp in the full flowering stage, it can be concluded that UAE is a superior extraction technique due to higher EY (17.54 compared to 14.24%), TP (1.5245 compared to 1.0476 mg GAE/mL), TF (0.6214 compared to 0.3584 mg CE/mL), and CBD (0.8827 compared to 0.4310 mg/mL). Also, when UAE is applied as an extraction technique, the time reduction is significant: 1 h for UAE compared to 24 h for conventional extraction.

**Figure 2:**
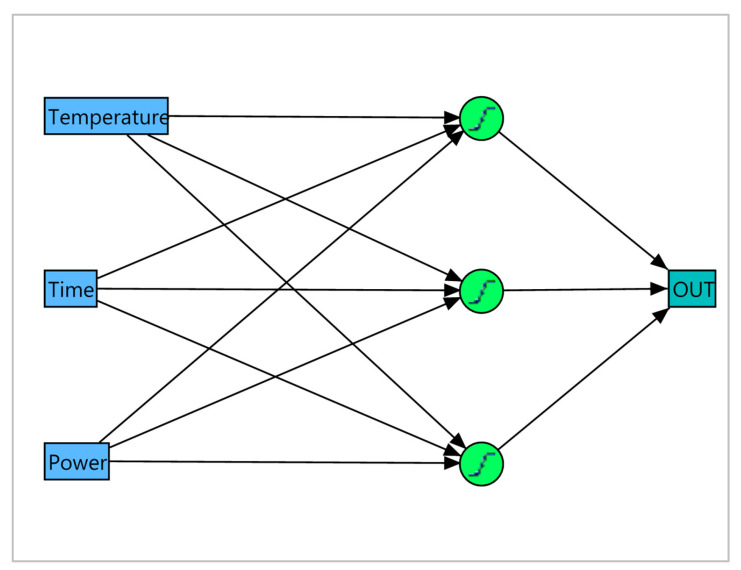
Artificial neural network (ANN) with three neurons at the hidden level.

ANN modeling and optimization highly depend on the construction of the hidden layer. The number of neurons in the hidden level is varied from 1 to 10 to avoid “over-fitting”. Based on the most acceptable statistics, ANNs with three neurons at the hidden level were chosen (Figure 2). The root-mean-square error (RMSE), mean absolute deviation (MAD), and the determination coefficient (R2) of the training and validation of each ANN are summarized in Table S4. The ANN models obtained provided an adequate fit for the experimental data due to their high coefficient of determination for training and validation (above 0.9 for TP, TF, CBD, and THC, and above 0.8 for EY) as well as their low RMSE and MAD. A comparison of the coefficient of determination of the two statistical approaches used indicates that ANN models have better fitting with experimental data. It was rather expected that ANN could provide a better fit due to its higher flexibility using different activation functions, while the RSM approach uses second-order polynomial functions. The better predictive capacity of the ANN models compared to RSM has been reported by many authors [49,50,51].

The influence of input variables on responses in ANN is present in Table S5. The main effect represents the influence of input variable alone, without combination with other inputs, while the total effect represents the influence of the input variables alone and in combination with other inputs. The sensitive analysis shows that the UAE parameter with the most influence on EY, TP, and TF was extraction temperature, while extraction time was the UAE parameter with the most influence on the CBD and THC. Ultrasound power has the lowest influence on UAE. The same results can be seen from the 3D plots present in Figure S3. A similar observation was noticed with RSM, where the temperature was the parameter which affected all responses, while power had an influence only on EY. Interactions between temperature and time have an influence on TF, CBD, and THC.

The optimal conditions obtained in ANNs for each response are presented in Table 6.

The confirmation of the predictive capacity of ANNs was observed through the optimal condition obtained by RSM. Based on the defined optimal conditions for TPs and TFs by RSM, ANNs predicted values for responses were as follows: 18.3516%, 1.5073 mg GAE/mL, 0.7893 mg CE/mL, 0.8401 mg/mL, and 0.0427 mg/mL for EY, TP, TF, CBD, and THC, while experimental results were 18.4600%, 1.5128 mg GAE/mL, 0.7895 mg CE/mL, 0.8565 mg/mL, and 0.0429 mg/mL, respectively.

## 3. Materials and Methods

### 3.1. Herbal Material

In 2017, the monoecious industrial hemp cultivar Helena was grown as a commercial crop and produced with technology recommended by Bócsa and Karus [52] at the experimental field in Bački Petrovac (45°20′ N 19°35′ E), Institute of Field and Vegetable Crops, Novi Sad, Serbia. Sampling was performed twice—during the beginning of crop flowering (beginning of the flowering stage) and 20 days later, when more than 70% of crop plants were in full flowering (full flowering stage)— following the field sampling protocol described previously by Drinić et al. [23].

The sampled plant material was air-dried to a residual humidity (less than 12%) at an ambient temperature, after which parts of the stem and grain were removed from the laboratory sample (which contained leaves, blossoms, small structural parts of the inflorescence, and bracts) manually with the aid of test sieves (mesh 1.5 mm). Laboratory samples were then ground in a domestic blender. The average particle size of the grind herbal material (0.4378 mm) was analyzed using a sieve set (CISA Cedaceria Industrial, Barcelona, Spain).

### 3.2. Chemicals

Folin–Ciocalteu, as well as 1,1-diphenyl-2-picryl-hydrazyl-hydrate (DPPH), were purchased from Sigma (Sigma-Aldrich GmbH, Steinheim, Germany). Both standard compounds, (±)-catechin and gallic acid, were purchased from Sigma (St. Louis, MO, USA). Potassium ferricyanide was purchased from Merck (Darmstadt, Germany). All other chemicals used in this study were of analytical reagent grade.

### 3.3. Conventional Extraction Procedure

The conventional extraction of grind hemp areal parts was performed at room temperature for a total duration of 24 h. The grind herbal material-to-solvent ratio was 1:10. Distillate water and ethanol/water mixtures (30, 50, 70, and 90% ethanol) were used as the extraction solvents. Extraction was followed via filtration through filter paper with pore sizes of 4–12 μm under the vacuum. Furthermore, to avoid the destabilization of the obtained liquid fraction/extract, they were collected into adequate glass volume flasks and stored in the freezer. The extraction yield has been expressed as a percentage (%). All experiments were conducted in the three replications, while the results are expressed as mean values.

### 3.4. UAE Procedure and Experimental Plan of Optimization

A sonication water bath (EUP540A, EU instruments, Paris, France) with a fixed frequency of 40 kHz was used for the extraction of hemp areal parts in the full flowering stage. In all experimental runs, the grind herbal material-to-solvent ratio was 1:10, where 50% ethanol was applied as an extraction solvent. The glass flasks were filled with the extraction mixture (grind herbal material and solvent). The flasks were supplied with the condenser, which was positioned in the same distance from the transducer in each extraction run. No additional agitation was applied. The extraction parameters (ultrasonic power, extraction time, and extraction temperature) were controlled from the panel of the instrument. Extraction was conducted according to experimental plan presented in Table 3.

Like the case of conventional extraction, to avoid the destabilization of the obtained liquid fraction/extract, they were collected into the adequate glass volume flasks and stored in the freezer at an adequate storage temperature. The EY was determined, in the same way as in the case of the conventional extraction, and expressed as a percentage (%). All experiments were performed in three replications, while the results are expressed as mean values.

Response surface methodology (RSM) was applied to investigate and optimize the effect of different input variables in UAE (extraction time, extraction temperature, and ultrasonic power) on the output parameters (extraction yield, total phenol content, total flavonoid content, CBD content, and THC content). For the experimental design, a Box–Behnken design (BBD) with three numerical factors on three levels was selected. The BBD consisted of 17 randomized runs with 5 replicates at the central point. A set of 17 experiments were generated with a random combination of independent variables. In order to normalize the parameters, each actual value of an independent variable was coded according to Equation (1) [53]:

(1) $$X=\frac{x_{i}-x_{0}}{∆x}$$

where X is the coded value, x_i_ is the corresponding actual value, x_0_ is the actual value in the center of domain, and Δx is the increment of xi corresponding to a variation of 1 unit of X. The coded values of the independent variables allow all to affect the response more evenly, and thus, the units of parameters are irrelevant [53]. The actual and coded values of the input variables are shown in Table 7 [53]. The actual and coded values of the input variables are shown in Table 7.

According to Bezerra et al. [54], in order to best describe the relationship between the input and output variables, the responses must be fitted with the following second-order polynomial Equation (2):

(2) $$Y=\beta_{0}+\sum_{i=1}^{3}\beta_{i}X_{i}+\sum_{i=1}^{3}\beta_{ii}X_{i}^{2}+\sum_{i<j=1}^{3}\beta_{ij}X_{i}X_{j}$$

where Y represents the response variable, X_i_ and X_j_ are the input variables, and *β*_0_, *β*_i_, *β*_ii_, and *β*_ij_ are the regression coefficients for linear, quadratic, and interaction terms, respectively.

Optimal extraction conditions were determined separately according to the total phenol and total flavonoid content, as well as the content of CBD and THC: (a) The optimization according to the total phenol and total flavonoid content of the extraction conditions was determined with respect to the maximum content of total phenol and total flavonoid. (b) The optimization, according to the content of CBD and THC, was performed in two cases. In both cases, the optimization was performed with respect to the maximum content of CBD, while the content of THC in the first case was set up on minimum values, and in the second case, it was not taken into account.

The analysis of the experimental data was conducted with Design-Expert V. 7 Trial (Stat-Ease, Minneapolis, MN, USA). Multiple regression analysis was employed to analyze the experimental data. The regression coefficients of linear, interaction, and quadratic terms were evaluated through analysis of variance (ANOVA). The adequacy of the models was evaluated by the coefficient of determination (R2), coefficient of variance (CV), and *p* values for the model and lack-of-fit testing. The validity of the optimal process condition was experimentally confirmed.

An artificial neural network (ANN) has also been applied as a statistical method for modeling and optimizing the UAE. The data shown in Table 3 were used for the training and validation of the neural network. JMP 14 Trial (SAS Institute, Inc., Cari, NC, USA) was employed to develop multilayer perceptron networks. For each investigated response, a separate ANN was developed, which consisted of three layers: an input layer, with three neurons representing the input variables (extraction time, extraction temperature, and ultrasonic power), a hidden layer, and an output layer, with one neuron representing the output variable (extraction yield, total phenol content, total flavonoid content, CBD content, or THC content). The optimization of the ANN model was conducted by determining the appropriate number of neurons in the hidden layer by using a trial-and-error approach in order to obtain the lowest root-mean-square error (RMSE) and the highest determination coefficient (R^2^) of training and validation. As the activation function between the input and hidden layer, the hyperbolic tangent function was used. The K-fold cross-validation procedure was applied for the validation and testing of developed ANNs because it is suitable for limited data samples. The optimal extraction conditions by ANNs were determined separately for each response.

### 3.5. Characterization of Hemp Extracts

#### 3.5.1. Total Phenol Content

To determine the concentration of the total phenolic compounds (TPs) in the obtained liquid extracts, the conventional analysis method—the Folin–Ciocalteu spectrophotometric procedure—was applied [55]. In this case, the absorbance of the investigated samples was measured at 750 nm (6300 Spectrophotometer, Jenway, UK). Gallic acid was used as a standard compound for the preparation of the calibration curve. The content of total phenols in the investigated samples has been expressed as mg of gallic acid equivalent per mL liquid extract (mg GAE/mL extract). All experiments were replicated three times, and the results were expressed as mean values.

#### 3.5.2. Total Flavonoids Content

To determine the concentration of the total flavonoid compounds (TFs), another conventional spectrophotometric method that uses aluminum chloride was applied [56]. The sample absorption was measured at the wavelength range of 510 nm. Catechin was used as a standard compound for the preparation of the calibration curve. The results were expressed as mg of catechin equivalent per mL liquid extract (mg CE/mL extract). All experiments were replicated three times, and the results were expressed as mean values.

#### 3.5.3. HPLC Analysis of Polyphenolic Constituents

Phenolic compounds were analyzed using the HPLC method described by Mišan et al. [57]. The analysis was performed by using an Agilent 1200 series (Agilent Technologies, Santa Clara, CA, USA) liquid chromatograph equipped with a diode array detector (DAD), a binary pump, an online vacuum degasser, Chemstation Software version B.03.01 (Agilent Technologies, Santa Clara, CA, USA), an autosampler, and a column (4.6 mm by 50 mm, 1.8 μm packing, Agilent Technologies, Eclipse XDB-C18), at a flow rate of 1 mL/min. Solvent gradient was performed by varying the proportion of solvent A (methanol) to solvent B (1% (*v*/*v*) formic acid in water). The elution conditions were as follows: initial 10% solvent A (methanol); 0–10 min, 10–25% solvent A; 10–20 min, 25–60% solvent A; and 20–30 min, 60–70% solvent A. The injection was performed automatically using an autosampler, and the volume of the tested samples and standards was 5 μL. The spectra were recorded in the range of 210–400 nm, and chromatograms were plotted at 280, 330, and 350 nm. The content of the phenolic compounds was determined from their calibration curves, and expressed in mg per mL of extract. Calibration curves were plotted on the basis of five calibration points, and the determination coefficients were calculated. For all the compounds investigated, the determination coefficient was higher than 0.9995.

#### 3.5.4. GC/MS Analysis of CBD and THC

The content of CBD and THC in the obtained liquid extracts was determined by GC-MS analysis. Absolute methanol (2.5 mL) was added to 0.5 mL of extract and then shaken, and after that, it was centrifuged at 10,000 rpm for 5 min. The supernatant was transferred to a GC vial. The decarboxylation step of the acid form of CBD and THC happened in the GC-MS inlet at a temperature of 280 °C. Analysis of cannabinoids was performed on Agilent 6890N GC equipped with a mass spectrum (MS) detector, Agilent 5975B. The separation was performed on a fused silica capillary column (HP-5MS, 30 m × 0.25 mm i.d., and 0.25 µm film thickness). Helium was used as carrier gas at a constant flow of 1 mL/min. The temperature program was as follows: the initial temperature of 200 °C was held for 2 min, then increased to 240 °C at a rate of 10 °C/min, and held for 10 min. The injector and detector temperatures were set at 280 and 230 °C, respectively. The injected sample volume was 1.5 μL, and the split ratio was 1:20. Individual analytical standards for cannabidiol (CBD), cannabigerol (CBG), and cannabinol (CBN) were used for the calibration. The quantitation of THC was performed with the CBN analytical standard in accordance with the method given by Poortnman-van der Meer et al. [58].

## 4. Conclusions

The aim of this study was to investigate the potential of conventional and ultrasound-assisted extraction to be applied for the preparation of hemp extracts from areal parts cv. Helena, where the targeted compounds of interest were phenolics and cannabinoids. UAE showed advantages over the conventional extraction method, which are reflected in the reduction in extraction time and in the higher content of target compounds. The extracts obtained by UAE had a higher content of phenolics and cannabinoids, as well as a higher extraction yield. UAE had a positive effect on the isolation of protocatechuic acid, vanillic acid, ferulic acid, sinapic acid, rutin, and naringenin. Total phenol content was about 50% higher in UAE extracts, while total flavonoid content was about 2-fold higher in UAE extracts. The UAE-obtained extract has a higher content of CBD, while THC content was similar regardless of which extraction method was used. Comparing the plant material in different stages of growth, it can be concluded that hemp aerial parts at the beginning of flowering represent a good source of the phenolic compound with sinapic acid and apigenin as dominant facets, while hemp aerial parts in the full flowering stage represent a good source of cannabinoids. When conventional extraction is used to obtain hemp extract, 50% ethanol as solvent should be used. The UAE values of the investigated parameters which should be used for the extract obtained from hemp at the full flowering stage, that is rich in phenol compounds, are as follows: an extraction temperature of 68.8 °C, an extraction time of 54.8 min, and an ultrasonic power density of 60 W/L. In parallel, extract rich in CBD can be obtained when following the conditions applied: an extraction temperature of 79.4 °C, an extraction time of 31.6 min, and an ultrasonic power density of 60 W/L. Also, this study indicates that the commercial crop Helena is a good source of CBD, which is the main active compound in hemp.

## Figures and Tables

**Table 1:** Effect of extraction solvents on extraction yield (EY), total phenols (TPs), and total flavonoids (TFs) content of hemp.

Solvent	Hemp at the Beginning of the Flowering Stage			Hemp in the Full Flowering Stage		
	EY [%]	TP [mg GAE/mL]	TF [mg CE/mL]	EY [%]	TP [mg GAE/mL]	TF [mg CE/mL]
90% ethanol	8.18 ± 0.41 e	0.5962 ± 0.0298 e	0.2370 ± 0.0119 ef	7.54 ± 0.38 e	0.5535 ± 0.0277 b	0.3169 ± 0.02 b
70% ethanol	12.84 ± 0.64 d	1.7045 ± 0.0853 a	0.4451 ± 0.0223 bc	11.66 ± 0.58 c	1.0386 ± 0.0519 a	0.3323 ± 0.02 ab
50% ethanol	16.96 ± 0.85 bc	1.2565 ± 0.0629 b	0.4817 ± 0.0241 b	14.24 ± 0.71 b	1.0476 ± 0.0524 a	0.3584 ± 0.02 a
30% ethanol	17.34 ± 0.87 bc	1.0981 ± 0.0549 c	0.3022 ± 0.0151 d	15.22 ± 0.76 ab	1.0206 ± 0.0510 a	0.2174 ± 0.01 d
Water	17.94 ± 0.90 ab	0.5759 ± 0.0288 e	0.3950 ± 0.0198 c	15.28 ± 0.76 ab	0.4996 ± 0.0250 b	0.1674 ± 0.01 e

Different letters within the column represent a statistically significant difference between the tested qualitative parameters of the extracts, based on Tukey’s test *p* < 0.05; mg GAE/mL—mg of gallic acid equivalent per mL liquid extract; mg CE/mL—mg of catechin equivalent per mL liquid extract.

**Table 2:** Qualitative and quantitative profiles of individual phenolic compounds (in µg/mL) detected in the extracts produced from hemp areal parts at the beginning of the flowering stage and in the full flowering stage.

	Solvent	Protocatechuic Acid	Vanillic Acid	Syringic Acid	Epicatechin	Ferulic Acid	Sinapic Acid	Isovitexin	Rutin	Cinnamic Acid	Naringenin	Apigenin
hemp areal parts at the beginning of the flowering stage	90% Ethanol	1.09	0.45	nd ^a^	nd	2.57	1.13	1.14	4.13	0.81	1.21	0.31
	70% Ethanol	1.97	1.55	1.72	7.38	1.33	16.36	3.28	6.77	1.37	nd	7.73
	50% Ethanol	1.50	1.44	nd	nd	3.61	30.02	1.84	0.19	1.44	nd	8.81
	30% Ethanol	1.66	0.63	nd	nd	2.49	3.21	0.92	0.19	1.38	33.16	4.16
	Water	0.36	nd	0.42	nd	1.36	2.81	nd	nd	1.71	2.14	2.01
hemp areal in the full flowering stage	90% Ethanol	2.50	1.07	nd	nd	nd	4.02	0.64	2.28	7.32	1.60	0.31
	70% Ethanol	1.24	1.53	4.77	3.59	nd	1.19	Nd	2.63	8.84	1.51	0.96
	50% Ethanol	nd	1.26	nd	nd	nd	1.03	6.21	2.74	3.28	1.33	1.10
	30% Ethanol	nd	0.58	nd	nd	nd	nd	Nd	nd	nd	1.70	0.98
	Water	nd	nd	nd	nd	nd	1.46	0.98	nd	nd	1.46	0.96

^a^ nd—not detected.

**Table 3:** Box–Behnken experimental design with ultrasound-assisted extraction parameters and experimentally obtained values of extraction yield (EY), total phenol (TP), total flavonoid (TF), cannabidiol (CBD), and tetrahydrocannabinol (THC).

	Independent Variables			Responses				
Run	Extraction Temperature (°C)	Extraction Time [min]	Ultrasonic Power Density (W/L)	EY [%]	TP [mg GAE/mL Extract]	TF [mg CE/mL Extract]	CBD [mg/mL]	THC [mg/mL]
1	60	40	42	13.34	1.3050	0.6391	0.7604	0.0439
2	60	20	60	14.78	1.3612	0.5405	0.7860	0.0442
3	80	20	42	15.88	1.4145	0.6722	0.8259	0.0455
4	60	40	42	14.74	1.2966	0.6426	0.7672	0.0444
5	60	60	60	17.02	1.4258	0.6749	0.7912	0.0447
6	40	60	42	12.66	1.1590	0.5137	0.6924	0.0425
7	80	40	60	17.04	1.4314	0.5808	0.8752	0.0458
8	40	20	42	10.20	1.0552	0.3793	0.6158	0.0412
9	60	40	42	13.66	1.3752	0.5620	0.7154	0.0431
10	40	40	60	11.72	1.2208	0.4680	0.6712	0.0421
11	60	40	42	17.54	1.4763	0.5925	0.7289	0.0437
12	60	20	24	14.10	1.3949	0.5378	0.6966	0.0428
13	80	40	24	14.94	1.4987	0.6543	0.8602	0.0453
14	60	60	24	12.16	1.3780	0.5656	0.7329	0.0440
15	40	40	24	10.00	1.1534	0.4563	0.6698	0.0420
16	80	60	42	16.56	1.7795	0.5459	0.7612	0.0440
17	60	40	42	14.68	1.2882	0.5262	0.7946	0.0441

mg GAE/mL—mg of gallic acid equivalent per mL liquid extract; mg CE/mL—mg of catechin equivalent per mL liquid extract.

**Table 4:** Estimated optimal conditions for the investigated range and predicted values of investigated responses (extraction yield (EY), total phenol (TP), total flavonoid (TF), cannabidiol (CBD), and tetrahydrocannabinol (THC)).

	Optimal Conditionals			Predicted Values				
Target	Extraction Temperature (°C)	Extraction Time [min]	Ultrasonic Power Density (W/L)	EY [%]	TP [mg GAE/mL Extract]	TF [mg CE/mL Extract]	CBD [mg/mL]	THC [mg/mL]
TP max TF max	68.8	54.8	60	17.54	1.5245	0.6214	0.8077	0.0448
CBD max THC none	79.4	31.6	60	16.85	1.4435	0.6092	0.8827	0.0460
CBD max THC min	55.4	38.2	24	13.43	1.2370	0.5368	0.7339	0.0434

mg GAE/mL—mg of gallic acid equivalent per mL liquid extract; mg CE/mL—mg of catechin equivalent per mL liquid extract.

**Table 5:** The profile of individual phenolic compounds (in μg/mL) detected in the hemp extracts produced on calculated optimal parameters.

Sample	Protocatechuic Acid	Vanillic Acid	Ferulic Acid	Sinapic Acid	Isovitexin	Rutin	Cinnamic Acid	Naringenin	Apigenin
Hemp aerial parts at the beginning of the flowering stage	1.34	1.95	3.36	25.00	6.56	1.52	1.55	6.50	2.00
Hemp aerial parts in the full flowering stage	1.28	2.22	1.52	10.04	1.04	3.92	2.04	2.50	1.28

**Table 6:** Optimal conditions obtained in the artificial neural network (ANN) for the investigated responses (extraction yield (EY), total phenol (TP), total flavonoid (TF), cannabidiol (CBD), and tetrahydrocannabinol (THC)).

	Extraction Temperature (°C)	Extraction Time [min]	Ultrasonic Power Density (W/L)	Predicted Value
EY [%]	55.03	70.15	60	18.36
TP [mg GAE/mL extract]	60	80	41.53	1.7791
TF [mg CE/mL extract]	60	79.12	60	0.9108
CBD [mg/mL]	44.62	80	60	1.1347
THC [mg/mL]	20	80	60	0.1488

mg GAE/mL—mg of gallic acid equivalent per mL liquid extract; mg CE/mL—mg of catechin equivalent per mL liquid extract.

**Table 7:** Actual and coded valued of input variables.

Input Variable	Values of Input Variables					
	Coded	Actual	Coded	Actual	Coded	Actual
Extraction temperature [°C]	−1	40	0	60	1	80
Extraction time [min]	−1	20	0	40	1	60
Ultrasonic power density [W/L]	−1	24	0	42	1	60

## Data Availability

Data are contained within the article and Supplementary Materials.

## Supplementary Materials

The following supporting information can be downloaded at: https://www.mdpi.com/article/10.3390/plants14050816/s1, Tables S1–S5 and Figures S1–S3.
