# Supplementary material for: Maximizing Cannabinoid and Polyphenol Extraction from Industrial Hemp (Cannabis sativa L. cv. Helena) Areal Parts: A Comparative Study of Ultrasound-Assisted and Conventional Methods at Two Harvest Stages
Source: Plants (Basel). 2025 Mar 5;14(5):816. doi: 10.3390/plants14050816 (PMC11902237; doi:10.3390/plants14050816)
Supplement: Supplementary file 1 [file plants-14-00816-s001.zip › plants-3448637-supplementary.pdf]

Supplementary material

Table S1. Results of ANOVA for extraction yield (EY), total phenol (TP), total flavonoid (TF), cannabinoid (CBD), and tetrahydrocannabinol (THC).

Source	Sum of squares	DF	Mean square	F value	p value
EY					
Model	71.94	9	7.99	4.51	0.0297
Residual	12.39	7	1.77		
Lack of fit	1.44	3	0.48	0.17	0.9083
Pure error	10.96	4	2.74		
Total	84.33	16			
R^2=0.8530					
CV=9.38%					
TP					
Model	35.99	9	4	4	0.0405
Residual	6.99	7	1		
Lack of fit	4.47	3	1.49	2.36	0.2129
Pure error	2.53	4	0.63		
Total	42.98	16			
R^2=0.8373					
CV=7.38%					
TF					
Model	8.87	9	0.99	4.11	0.0378
Residual	1.68	7	0.24		
Lack of fit	0.68	3	0.23	0.9	0.5144
Pure error	1	4	0.25		
Total	10.54	16			

R^2=0.8409					
CV=8.71%					
CBD					
Model	0.07	9	$7,77 \times 10^{-3}$	6.88	0.0094
Residual	$7,91 \times 10^{-3}$	7	$1,13 \times 10^{-3}$		
Lack of fit	$3,93 \times 10^{-3}$	3	$1,31 \times 10^{-3}$	1.32	0.3849
Pure error	$3,98 \times 10^{-3}$	4	$9,94 \times 10^{-4}$		
Total	0.078	16			
R^2=0.8984					
CV=4.48%					
THC					
Model	$2,50 \times 10^{-5}$	9	$2,78 \times 10^{-6}$	10.45	0.0027
Residual	$1,86 \times 10^{-6}$	7	$2,66 \times 10^{-7}$		
Lack of fit	$9,28 \times 10^{-7}$	3	$3,09 \times 10^{-7}$	1.33	0.3825
Pure error	$9,32 \times 10^{-7}$	4	$2,33 \times 10^{-7}$		
Total	$2,68 \times 10^{-5}$	16			
R^2=0.9307					
CV=1.18%					

R^2 - coefficient of determination, CV - coefficient of variation

Table S2. Corresponding p values of linear, interaction and quadratic terms of regression coefficients obtained for selected response variables (extraction yield (EY), total phenol (TP), total flavonoid (TF), cannabinoid (CBD), and tetrahydrocannabinol (THC)).

**Term**	**Response**				
	**EY**	**TP**	**TF**	**CBD**	**THC**
Linear					
X_1	**0.0012**	**0.001**	**0.0025**	**0.0002**	**<0.0001**
X_2	0.3911	0.1104	0.2587	0.5929	0.3254
X_3	**0.0418**	0.9618	0.7278	0.1281	0.1117
Interaction					
X_1X_2	0.525	0.2328	**0.0324**	0.0737	**0.0335**
X_1X_3	0.8905	0.5218	0.4136	0.8443	0.7645
X_2X_3	0.1602	0.6959	0.3123	0.6589	0.5359
Quadratic					
X_1^2	0.1567	0.5569	0.0645	0.7239	0.2219
X_2^2	0.9271	0.5097	0.6185	0.1954	0.4558
X_3^2	0.6178	0.8763	0.9892	0.2246	0.3079

X_1-extraction temperature, X_2-extraction time, X_3-ultrasonic power density, $p<0.01$ remarkably significant, $0.01<p<0.05$ highly significant, $0.05<p<0.1$ significant

Table S3. Mathematical equation describing the model for extraction yield (EY), total phenols (TP), total flavonoids (TF), cannabidiol (CBD) and tetrahydrocannabinol (THC) content.

Response	Mathematical equation
EY	$Y = 14.79 + 2.48X_1 + 1.17X_3$
TP	$TP = 13.48 + 1.92X_1$
TF	$TF = 5.92 + 0.79X_1 - 0.65X_1X_2 - 0.52{X_1}^2$
CBD	$CBD = 0.75 + 0.084X_1 - 0.035X_1X_2$
THC	$THC = 0.044 + 1.616X_1 - 6.802 * 10^{-4}X_1X_2$

X_1-extraction temperature, X_2-extraction time, X_3-ultrasonic power density

Table S4. The root-mean-square error (RMSE), mean absolute deviation (MAD), and the determination coefficient (R^2) of the training and validation of artificial neural networks (ANNs) for extraction yield (EY), total phenol (TP), total flavonoid (TF), cannabinoid (CBD), and tetrahydrocannabinol (THC).

**Source**	**Training**	**Validation**
EY		
R^2	0.8722	0.8082
RMSE	0.8599	0.7932
MAD	0.5094	0.5443
TP		
R^2	0.9345	0.9167
RMSE	0.0447	0.0301
MAD	0.0252	0.0255
TF		
R^2	0.9316	0.9312
RMSE	0.0215	0.0298
MAD	0.0108	0.0184
CBD		
R^2	0.9574	0.9539
RMSE	0.0183	0.0199
MAD	0.0123	0.0178
THC		
R^2	0.9947	0.9683
RMSE	0.0002	0.0011
MAD	0.0001	0.0007

Table S5. The main and total effects of input variables (extraction time, extraction temperature, and power) on output variables (extraction yield (EY), total phenol (TP), total flavonoid (TF), cannabinoid (CBD), and tetrahydrocannabinol (THC).

**Source**	**Main effects**	**Total effects**
EY		
Extraction time	0.279	0.279
Extraction temperature	0.492	0.492
Power	0.186	0.186
TP		
Extraction time	0.218	0.218
Extraction temperature	0.399	0.399
Power	0.136	0.136
TF		
Extraction time	0.186	0.186
Extraction temperature	0.521	0.521
Power	0.293	0.293
CBD		
Extraction time	0.472	0.472
Extraction temperature	0.343	0.343
Power	0.185	0.185
THC		
Extraction time	0.662	0.662
Extraction temperature	0.299	0.299
Power	0.039	0.039

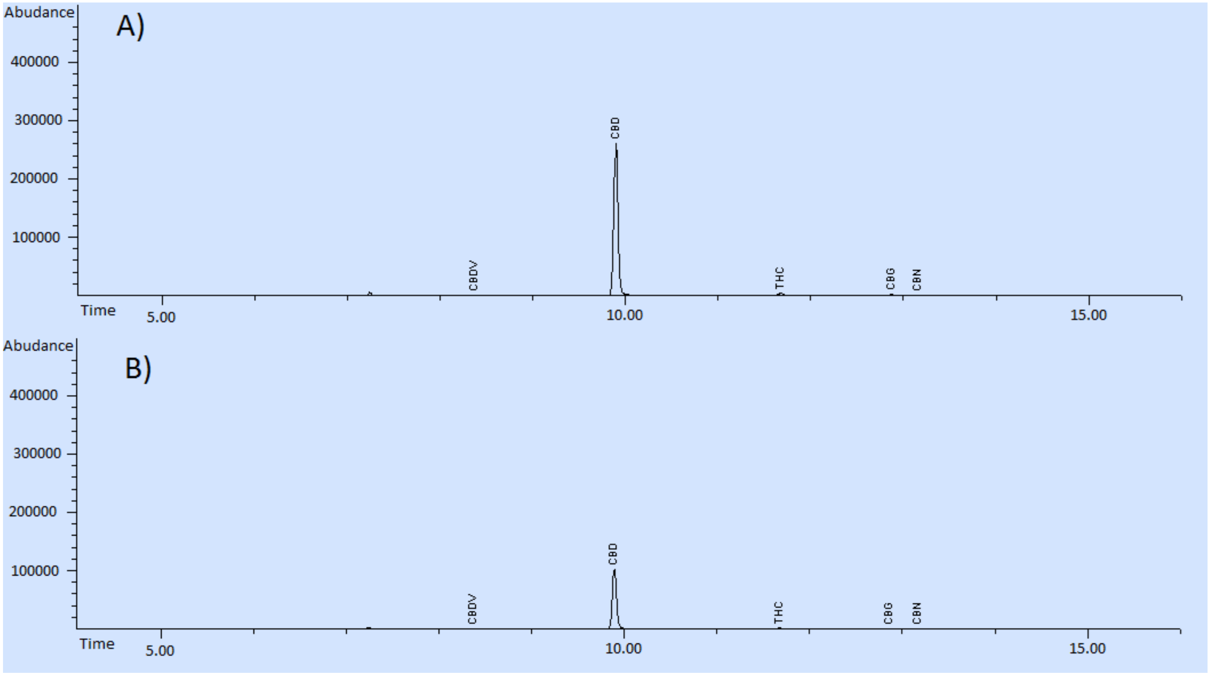

Figure S1. GC/MS chromatogram for: A) extract obtained by ultrasound-assisted extraction on optimal condition (extraction temperature 68.8°C, extraction time 54.8 min and ultrasound power density 60 W/L) and B) extract obtained by maceration with 50% ethanol.

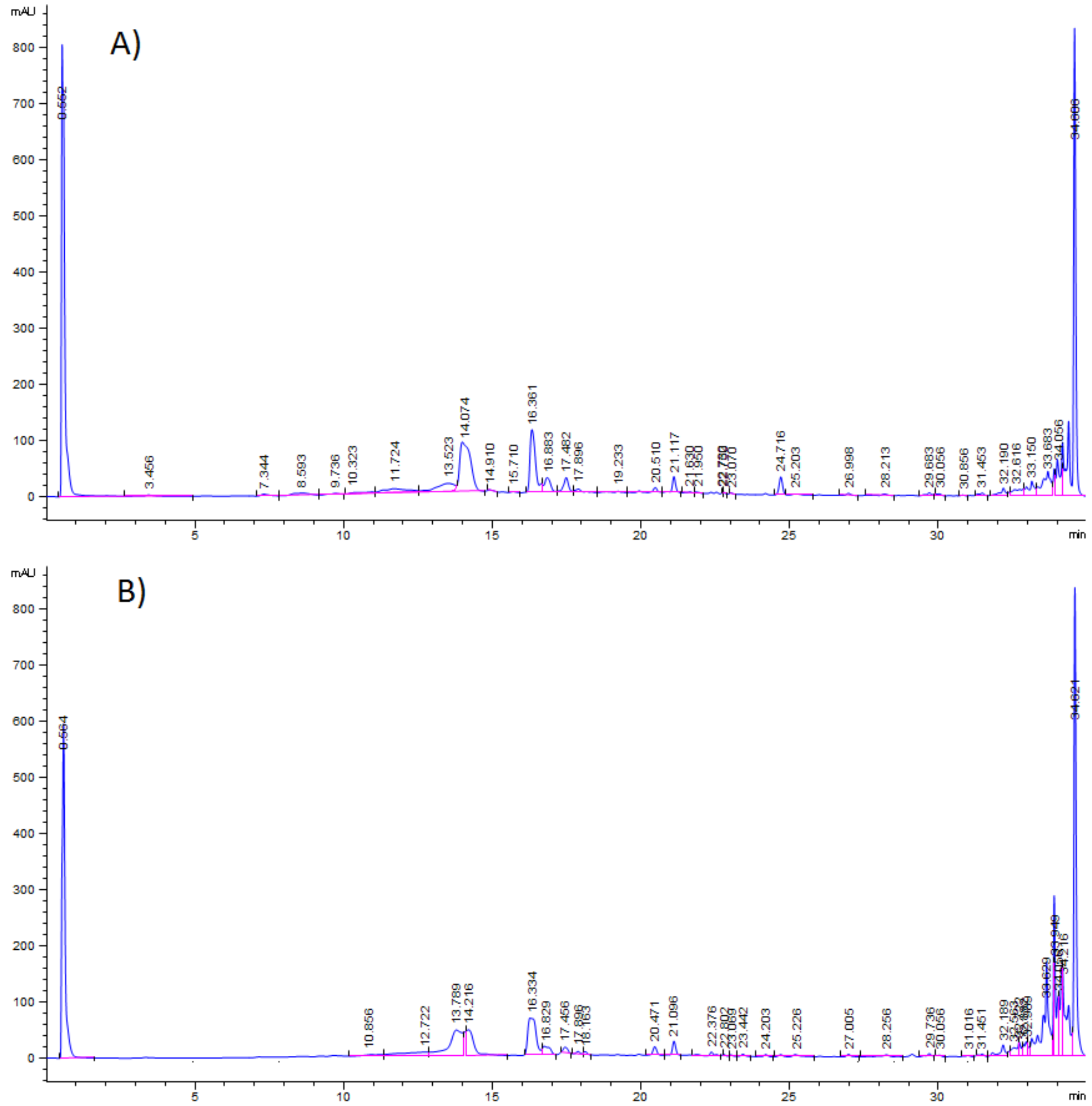

Figure S2. HPLC chromatogram for A) extract obtained by ultrasound-assisted extraction on optimal condition (extraction temperature 68.8°C, extraction time 54.8 min and ultrasound power density 60 W/L) and B) extract obtained by maceration with 30% ethanol.

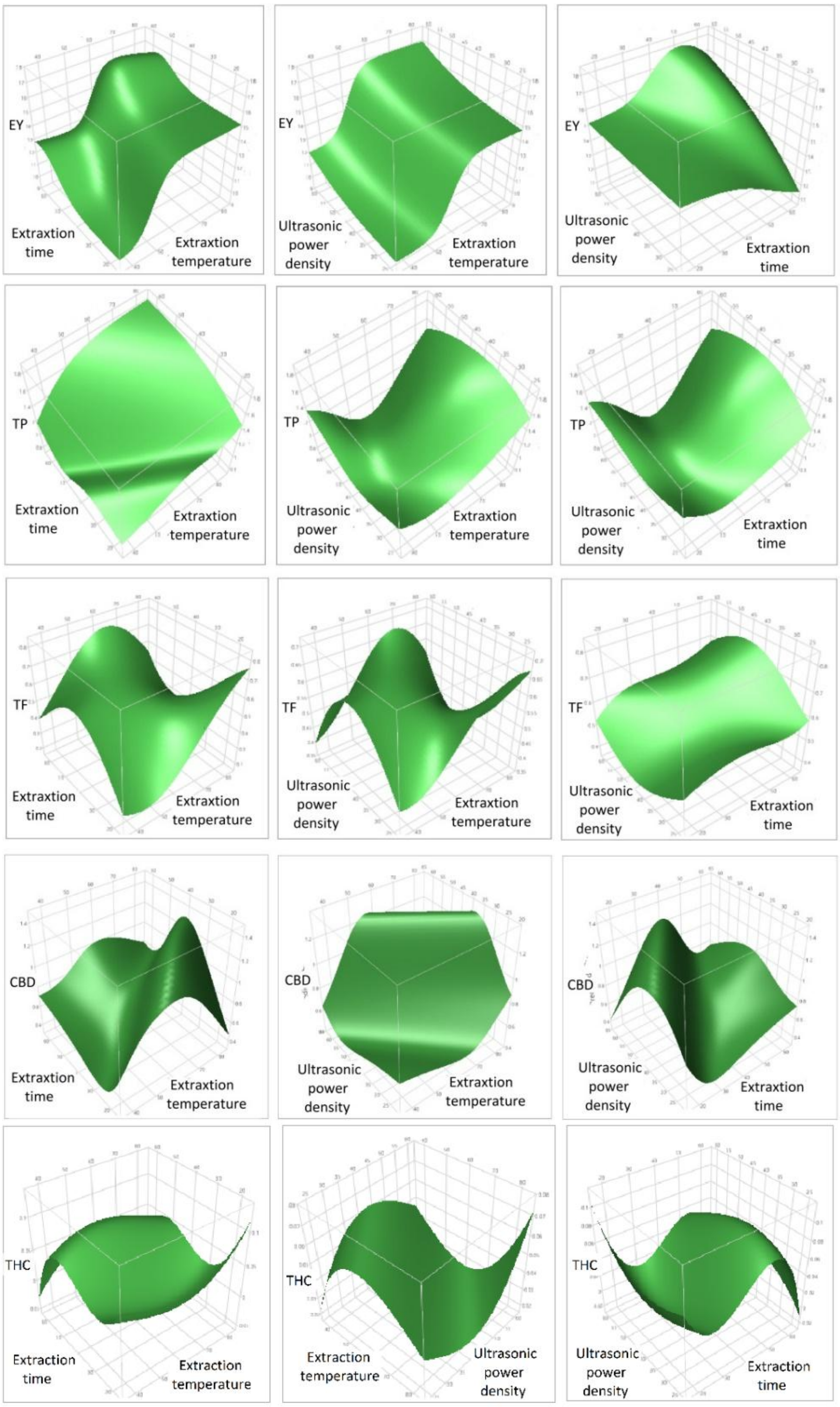

Figure S3. 3D plots obtained by artificial neural network (ANN) showing combined effects of parameters on extraction yield (EY), total phenol (TP), total flavonoid (TF), cannabidiol (CBD), tetrahydrocannabinol (THC).
